# Clinical application of psychological intervention in perioperative patients with thoracic tumors

**DOI:** 10.12669/pjms.41.10.12395

**Published:** 2025-10

**Authors:** Kun Mi, Duo Zhang, Hefei Li, Wenhua Sang, Qiang Guo

**Affiliations:** 1 Kun Mi The Second Department of Affective Disorders, Hebei Provincial Mental Health Center, Baoding 071000, Hebei, China; 2 Duo Zhang Department of Thoracic Surgery, Affiliated Hospital of Hebei University, Baoding 071000, Hebei, China; 3 Hefei Li Department of Thoracic Surgery, Affiliated Hospital of Hebei University, Baoding 071000, Hebei, China; 4 Wenhua Sang The Second Department of Affective Disorders, Hebei Provincial Mental Health Center, Baoding 071000, Hebei, China; 5 Qiang Guo Department of Thoracic Surgery, Affiliated Hospital of Hebei University, Baoding 071000, Hebei, China

**Keywords:** Thoracic tumor, Psychological status, Perioperative period, Psychological intervention, Quality of life

## Abstract

**Objective:**

To investigate the anxiety and depression status of perioperative patients with thoracic tumors and evaluate the effects of psychological intervention.

**Methodology:**

This was a retrospective study. A total of one hundred patients with thoracic tumors admitted to the Thoracic Surgery Department of Affiliated Hospital of Hebei University from January 2023 to October 2024 were selected and divided into an observation group and a control group by the specific intervention administered. The control group received standard treatment, while the observation group received targeted psychological intervention in addition to standard treatment based on individual conditions. The two groups were compared in terms of anxiety and depression levels, patient compliance, sleep quality, quality of life, hospitalization satisfaction, and adverse reactions.

**Results:**

After the intervention, the scores on the Self-Rating Anxiety Scale, Self-Rating Depression Scale, and Pittsburgh Sleep Quality Index in the intervention group were significantly lower than those in the control group (*P*< 0.05, respectively). The intervention group also showed significant improvement in physical function, mental function, social function, and material living conditions compared to the control group (*P*< 0.05, respectively). The overall compliance rate in the intervention group was 86.00%, significantly higher than the 68.00% in the control group (*P*< 0.05). Moreover, patient satisfaction in the intervention group reached 92.00%, significantly higher than 76.00% in the control group (*P*< 0.05).

**Conclusion:**

Perioperative patients with thoracic tumors have a high incidence of anxiety and depression. Comprehensive and effective psychological intervention can effectively alleviate anxiety and depression, enhance patient compliance, improve sleep quality and overall quality of life, and increase hospitalization satisfaction.

## INTRODUCTION

The incidence of thoracic tumors has been increasing annually, making it one of the major threats to human health.^1^ Most thoracic tumors require surgical treatment, and during the perioperative period, patients not only endure physical distress caused by the disease but also face numerous psychological challenges. Under the dual impact of surgery and tumor diagnosis, most patients experience psychological reactions such as tension, anxiety, depression, pessimism, insomnia, and fear.^2^ These psychological burdens can also affect the immune, nervous, and endocrine systems, negatively impacting surgical outcomes, postoperative recovery, and disease prognosis.

In severe cases, these effects can accelerate disease progression and significantly reduce patients’ quality of life.^3^ Extensive research^4^,^5^ has confirmed that psychological intervention for patients with perioperative tumors effectively alleviates anxiety and depression, improves sleep quality, and facilitates postoperative recovery. Various psychological intervention approaches are increasingly applied in clinical practice to alleviate patients’ negative emotions, thereby exerting a positive impact on clinical treatment and prognosis. However, there are limited studies on the effects of psychological intervention specifically in perioperative patients with thoracic tumors. Therefore, this study aimed to investigate the psychological status of perioperative patients with thoracic tumors and evaluate the clinical effects of psychological intervention, providing a reference for clinical practice.

## METHODOLOGY

This was a retrospective study. A total of one hundred patients with thoracic tumors who underwent surgical treatment in the Thoracic Surgery Department of Affiliated Hospital of Hebei University from January 2023 to October 2024 were selected as research subjects. The patients were divided into an intervention group and a control group, with 50 cases in each group. The control group received standard treatment, while the intervention group received psychological intervention in addition to standard treatment. Psychological intervention was administered once before surgery and then once daily starting from the first postoperative day until discharge.

### Ethical Approval:

The study was approved by the Institutional Ethics Committee of Affiliated Hospital of Hebei University (No.: HDFYLL-KY-2023-082; Date: June 19, 2023), and written informed consent was obtained from all participants.

### Inclusion criteria:

- Clinically diagnosed with thoracic tumors and undergoing surgical treatment, aged ≥18 years.
- Complete clinical data with good communication ability.
- Voluntary participation in the study with signed informed consent.
- No significant neuropsychiatric symptoms, good compliance, and able to cooperate with the study.

### Exclusion criteria:

- Patients with severe hepatic or renal dysfunction or other organic diseases.
- Patients with other concomitant tumors.
- Patients unwilling to follow up or with psychiatric disorders or cognitive impairments.
- Patients with malignant tumors in other parts of the body and patients with metastatic liver cancer.

### Intervention Methods:

Standard treatment: After admission, patients received relevant clinical education at different treatment stages. Treatment methods were based on international and national guidelines. Psychological intervention: (1) Positive psychological suggestion: Sincere doctor-patient communication was established to understand patients’ inner feelings. Patient compliance was improved through persuasion, explanation, and reassurance. Patient education was carried out to provide information on the disease, treatment plan, prognosis, and necessity of treatment to eliminate doubts and encourage confidence in treatment. (2) Attention diversion: Verbal reassurance and physical contact were provided as appropriate, in order to address anxiety and agitation caused by postoperative pain and organ resection. Emotional expression and distraction methods were employed to reduce patients’ focus on body image changes and pain perception. (3) Emphasis on family support: Good communication with patients’ family members was established, informing them of the importance of cooperating with medical treatment. Family members were encouraged to provide a relaxed atmosphere and reliable emotional support to enhance patients’ sense of security and well-being.

### Outcome Measures:

Both groups completed the Self-Rating Anxiety Scale (SAS), Self-Rating Depression Scale (SDS), Pittsburgh Sleep Quality Index (PSQI), and Generic Quality of Life Inventory-74(GQOLI-74) within 24 hours of hospital admission. SAS, SDS, and PSQI were reassessed on the day of discharge, while GQOLI-74 was reassessed during the three-month follow-up. Additionally, patient compliance was classified as complete compliance (full cooperation with treatment), partial compliance (cooperation with some aspects of treatment), and non-compliance (resistance to treatment). The compliance rate was calculated as: Compliance rate = (Complete compliance rate + Partial compliance rate). Patient satisfaction before and after the intervention was assessed using the Patient Satisfaction Questionnaire Short Form (PSQ-18), categorized as “very satisfied”, “more than satisfied”, “satisfied”, “not sure”, and “dissatisfied”. The overall satisfaction rate was calculated as: Overall satisfaction rate = (Very satisfied cases + More than satisfied cases + Satisfied cases) / total cases × 100%. All the questionnaire was simply handed over to the participants to fill themselves. During the six-months follow-up of this study, the survival rate was 100%. Moreover, pre- and post-intervention data were extracted from standardized nursing assessment forms that are routinely completed at admission and follow-up visits.

### Statistical analysis:

Statistical analysis was performed using the software SPSS 22.0. Measurement data were expressed as mean ± standard deviation (*χ̅*±*s*), and comparisons before and after treatment were conducted using an independent sample t-test. Enumeration data were expressed as frequency and percentage (*n*[%]), and comparisons between groups were examined using the chi-square (*χ*²) test. A value of *P* < 0.05 was considered statistically significant.

## RESULTS

The intervention group consisted of 36 males and 14 females, aged 40-78 (57.32±8.06) years, including 40 cases of lung cancer and 10 cases of mediastinal tumor. The control group consisted of 34 males and 16 females, aged 38-77 (57.44±8.22) years, including 41 cases of lung cancer and 9 cases of mediastinal tumor. There was no statistically significant difference in sex, age, or disease type between the two groups (all *P* > 0.05), indicating comparability.

Before the intervention, there were no significant differences in SAS, SDS, and PSQI scores between the two groups (all *P* > 0.05). After the intervention, the intervention group had significantly lower SAS, SDS, and PSQI scores than the control group (*P* < 0.05, respectively) Table-I.

**Table-I:** Intergroup comparison of psychological and sleep status before and after intervention (*χ̅*±*S*)

Scale		Intervention group	Control group	t-value	P-value
SAS	Before intervention	52.96±6.93	52.84±6.05	0.09	0.93
	After intervention	43.06±5.61	47.82±5.14	4.42	0.00
SDS	Before intervention	54.52±5.52	55.16±6.11	0.55	0.58
	After intervention	41.40±6.22	45.80±5.32	3.80	0.00
PSQI	Before intervention	6.64±0.88	6.78±0.84	0.82	0.42
	After intervention	5.66±0.92	6.08±0.90	2.31	0.02

Before the intervention, there were no significant differences in physical function, mental function, social function, or material living conditions between the two groups (all *P* > 0.05). After the intervention, the intervention group showed significant improvement in these aspects compared to the control group (*P* < 0.05, respectively) Table-II.

**Table-II:** Intergroup comparison of GQOLI-74 score before and after Intervention (*χ̅*±*S*).

Subscale		Intervention group	Control group	t-value	P-value
Physical function	Before intervention	43.79±7.80	43.45±7.85	0.22	0.83
	After intervention	53.02±8.46	48.34±8.04	2.84	0.01
Mental function	Before intervention	42.49±7.67	44.16±7.96	1.06	0.29
	After intervention	50.78±7.38	46.71±8.28	2.59	0.01
Social function	Before intervention	54.34±6.56	54.96±6.96	0.46	0.65
	After intervention	62.57±8.11	58.57±8.65	2.39	0.02
Material living conditions	Before intervention	45.39±8.59	45.11±8.26	0.17	0.87
	After intervention	55.06±8.21	51.07±8.57	2.38	0.02

The overall compliance rate in the intervention group was 86.00%, significantly higher than the 68.00% in the control group (*P* < 0.05) Table-III.

**Table-III:** Intergroup Comparison of Patient Compliance Rates (n[%]).

Group	Complete compliance	Partial compliance	Non-compliance	Total compliance rate
Intervention group	26(52.00)	17(34.00)	7(14.00)	43(86.00)
Control group	19(38.00)	15(30.00)	16(32.00)	34(68.00)
*χ* ^2^				4.57
*P*-value				0.03

The patient satisfaction rate in the intervention group was 92.00%, significantly higher than the 76.00% in the control group (*P* < 0.05) Table-IV.

**Table-IV:** Intergroup comparison of patient satisfaction (n[%]).

Group	Very satisfied	More than satisfied	Satisfied	Not sure	Dissatisfied	Overall satisfaction
Intervention group	30(60.00)	10(20.00)	6(12.00)	3(6.00)	1(2.00)	46(92.00)
Control group	22(44.00)	9(18.00)	7(14.00)	7(14.00)	5(10.00)	38(76.00)
*χ* ^2^						4.76
*P*-value						0.03

## DISCUSSION

In this study, 72.00% of patients had an SAS score of ≥50, and 65.00% had an SDS score of ≥53, indicating significant anxiety and depression, with anxiety being the predominant symptom. Clinically, these patients exhibited evident restlessness, hypersensitivity, sleep disturbances, and pessimism. Survey results indicated that the primary factor affecting patients’ emotional state was concern about having cancer, followed by worries about high medical expenses, surgical efficacy, family support, and fear of death. This suggests that a lack of medical knowledge and excessive worry contribute significantly to patients’ psychological distress,^6^ ultimately influencing recovery and prognosis. Studies have shown that psychological intervention during the perioperative period can help patients establish new psychological balance and reduce psychological stress.^7^ Currently, most thoracic tumor cases are lung cancer or esophageal cancer. Many of these patients experience significant anxiety upon diagnosis due to the societal stigma associated with cancer. Concerns about whether to undergo surgery, postoperative outcomes, treatment costs, family attitudes, and fear of death further exacerbate their psychological distress.^8^-^10^

In this study, after the intervention, the SAS and SDS scores in the intervention group were significantly lower than those in the control group, and their scores for physical function, mental function, social function, and material living conditions improved significantly(*P*< 0.05, respectively). Psychological intervention is a structured process guided by psychological theories that systematically influence an individual’s psychological activities, personality traits, or psychological problems to achieve a desired outcome.^11^ Research confirms that psychological intervention positively impacts patients’ psychological well-being, physical health, and immune function.^12^ This indicates that psychological intervention effectively alleviates anxiety and depression and enhances the quality of life of patients with thoracic tumors.

This effect may be attributed to the monitoring of perioperative negative emotions and health education, which improve patients’ understanding of their condition and their ability to manage psychological distress. As a result, patients can better adapt to treatment and recovery, reducing perioperative psychological stress.^13^,^14^ During the perioperative period, patients experiencing immense psychological pressure are prone to tension and insomnia. Anxiety and depression further contribute to sleep disorders, affecting treatment outcomes and prognosis. Psychological intervention helps the body maintain a balanced state, alleviating stress and thereby improving sleep quality.^15^

The results of this study showed that after the intervention, the improvement in PSQI scores in the intervention group was significantly better than that in the control group (*P* < 0.05), consistent with findings from previous research. Low compliance during the perioperative period can negatively impact postoperative recovery. Studies^16^,^17^ have identified a lack of medical knowledge, severe postoperative pain, and negative emotions as high-risk factors for poor patient compliance. Psychological intervention and health education enhance patients’ understanding of their disease and the importance of treatment, fostering a more optimistic attitude and improving patient compliance.^18^

The results of this study revealed that the overall compliance rate in the intervention group was significantly higher than in the control group (*P* < 0.05), demonstrating that psychological intervention has a positive impact on both psychological and behavioral aspects, leading to better patient compliance. Furthermore, the findings indicated that patient satisfaction in the intervention group was significantly higher than in the control group (*P* < 0.05). This suggests that psychological intervention enhances perioperative satisfaction among patients with thoracic tumors, likely due to its role in boosting treatment confidence, fostering hope, and promoting overall well-being.^19^,^20^

### Limitations:

Despite its findings, this study has several limitations, including a modest sample size and a short follow-up period. Further research with a larger sample and extended follow-up is needed to explore the long-term impact of psychological intervention on perioperative care for patients with thoracic tumors.

## CONCLUSIONS

Perioperative patients with thoracic tumors have a high incidence of negative psychological states such as anxiety and depression. Implementing appropriate psychological intervention can significantly improve these negative emotions, enhance sleep quality and quality of life, and increase patient compliance and hospital satisfaction.

### Authors’ Contributions:

**KM** and **DZ:** Concept, study design, literature search and manuscript writing, approved the final manuscript and are responsible for the integrity of the study.

**HL** and **WS:** Data collection, data analysis, interpretation and critical review.

**QG:** Revision of manuscript and validation. Final Approval.
